# Quinoxaline 1,4-di-*N*-oxide Derivatives as New Antinocardial Agents

**DOI:** 10.3390/molecules29194652

**Published:** 2024-09-30

**Authors:** Isidro Palos, Alonzo González-González, Alma D. Paz-González, José C. Espinoza-Hicks, Debasish Bandyopadhyay, Norma Paniagua-Castro, Marlene S. Galeana-Salazar, Jorge Ismael Castañeda-Sánchez, Julieta Luna-Herrera, Gildardo Rivera

**Affiliations:** 1 Unidad Académica Multidisciplinaria Reynosa-Rodhe, Universidad Autónoma de Tamaulipas, Reynosa 88779, México; isi_palos@hotmail.com; 2 Laboratorio de Biotecnología Farmacéutica, Centro de Biotecnología Genómica, Instituto Politécnico Nacional, Boulevard del Maestro, s/n, Esq. Elías Piña, Reynosa 88710, México; agonzalezg1700@ipn.mx (A.G.-G.); apazg@ipn.mx (A.D.P.-G.); 3 Facultad de Ciencias Químicas, Universidad Autónoma de Chihuahua, Circuito Universitario S/N, Chihuahua 31125, México; jhicks@uach.mx; 4 School of Biological and Chemical Sciences (SIBCS), The University of Texas Rio Grande Valley, 1201 West University Drive, Edinburg, TX 78539, USA; debasish.bandyopadhyay@utrgv.edu; 5 School of Earth Environment & Marine Sciences (SEEMS), The University of Texas Rio Grande Valley, 1201 West University Drive, Edinburg, TX 78539, USA; 6 Departamento de Fisiología, Escuela Nacional de Ciencias Biológicas, Instituto Politécnico Nacional, Ciudad de México 11340, México; npaniagc@ipn.mx; 7 Departamento de Sistemas Biológicos, Universidad Autónoma Metropolitana-Xochimilco, Ciudad de México 09460, México; mar.galeana@hotmail.com (M.S.G.-S.); jcastanedas@correo.xoc.uam.mx (J.I.C.-S.); 8 Departamento de Inmunología, Escuela Nacional de Ciencias Biológicas, Instituto Politécnico Nacional, Ciudad de México 11340, México

**Keywords:** antibacterial, drugs, scaffold, *Nocardia*, quinoxaline 1,4-di-*N*-oxide

## Abstract

Mycetoma is currently considered as a neglected tropical disease. The incidence of mycetoma is unknown but most of the worldwide cases are present in the “mycetoma belt” including countries like Mexico, India, Senegal, and others. The treatment of mycetoma depends on the etiological agent responsible for the case. Treatment success reaches 60 to 90%; however, common treatment has been reported to be ineffective in some cases, due in part to resistance to the prescribed antibiotics. Therefore, it is necessary to develop new therapeutic options. In the past two decades, quinoxaline derivatives have shown relevance as antibacterial agents. Therefore, in this work, esters of quinoxaline 1,4-di-*N*-oxide derivatives were evaluated in vitro against the reference strain CECT-3052 from *N. brasiliensis*, six clinical isolates, and macrophages J774A.1 to determine their cytotoxicity and security index. Additionally, nine reference drugs were evaluated as controls. The results show that nine esters of quinoxaline 1,4-di-*N*-oxide derivatives had a minimum inhibitory concentration (MIC) < 1 µg/mL against the reference strain and four of them (**N-05**, **N-09**, **N-11**, and **N-13**) had an MIC < 1 µg/mL against the clinical isolates. Therefore, the scaffold quinoxaline 1,4-di-*N*-oxide could be used to develop new and more potent antinocardial agents.

## 1. Introduction

*Nocardia* are bacteria belonging to the Phylum Actinobacteria, Actinobacteria class, Actinomycetales order, and *Nocardia* genus [1]. Bacteria of the *Nocardia* genus are found ubiquitously in the environment and can cause a wide variety of diseases in humans, with clinical manifestations ranging from localized forms up to pulmonary or disseminated infections. *Nocardia brasiliensis* (*N. brasiliensis*) causes mainly skin to soft tissue infections with superficial cellulitis or abscess localized in the lower extremities in adults; these lesions are the result of traumatic cutaneous injury caused by elements contaminated with these microorganisms, mainly soil. A chronic stage of the skin infection is mycetoma, specifically known as actinomycetoma, to differentiate from mycetoma caused by filamentous fungus (eumycetoma); actinomycetoma has a slow progression and it is localized mainly in the foot; it produces tumefaction, subcutaneous nodules with the formation of fistulas, and pus discharge, with the presence of characteristics structures known as grains. The more serious forms of nocardiosis can compromise the lives of patients and occur mainly in immunocompromised patients [2,3].

The pharmacological treatment for mycetoma or mild forms of nocardiasis is based in a therapy with the drugs trimethoprim/sulfamethoxazole, while for more severe pulmonary forms, trimethoprim/sulfamethoxazole, linezolid, or minocicline is used, combined with amikacin or carbapenems or ceftriaxone [4,5]. However, in many cases, the treatments are not effective; therefore, it is necessary to have new therapeutic options against this bacterial genus.

**Figure 1:**
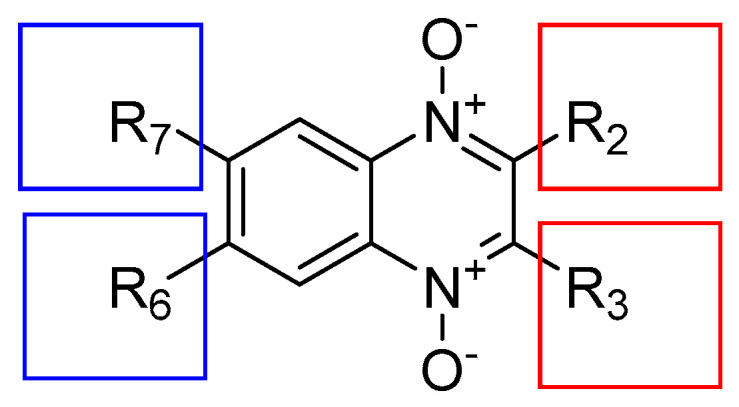
General structure of antibacterial compounds derived from quinoxaline 1,4-di-*N*-oxide.

On the other hand, quinoxalines (Figure 1) represent an important class of compounds that can be found in a variety of chemotherapeutic agents. The clinical importance of this class of compounds has stimulated the synthesis of new leading compounds that have a quinoxaline ring as a scaffold. Additionally, the oxidation of one or both nitrogen atoms on the quinoxaline ring to obtain *N*-oxide derivatives increases the diversity of biological activities. Interestingly, a similarity between quinoxaline and some antibacterial drugs has been reported [6], as well as the presence of the quinoxaline moiety in some broad-spectrum antibiotics (aquinomycin, levomycin, and actinoleutin), which suggests that quinoxaline could be used to develop new antibacterial agents.

In the past decade, quinoxaline 1,4-di-*N*-oxide derivatives with antibacterial activity have been reported [7,8,9]. However, Monge et al. (1995) observed that the loss of the two *N*-oxide groups generally leads to a loss of or decrease in antibacterial activity [10]. The first reports of quinoxaline 1,4-di-*N*-oxide derivatives with antibacterial activity confirmed that the presence of a methyl or methyl halogen group at 2-position on the quinoxaline ring (Figure 1) enhanced the antimicrobial activity. Carta et al. (2002) showed that the quinoxaline 1,4-di-*N*-oxide system is a good scaffold for antibacterial activity, which is favored by the presence of a methyl group at 3-position on the quinoxaline ring [11] (Figure 1). Zarranz et al. indicated that quinoxaline 1,4-di-*N*-oxide derivatives with a chlorine atom at 6- or 7-position on the quinoxaline ring show the best activity; however, electron-donor groups (CH_3_) reduce the activity [12]. In derivatives of 2-carbonylquinoxaline 1,4-di-*N*-oxide, with acetyl and benzoyl substituents at 2-position, they indicated that electron-acceptor groups at 6- and/or 7-position reduce the values of minimum inhibitory concentration (MIC) and half maximal inhibitory concentration (IC_50_); on the contrary, the electron-donor groups increase these values [13]. Subsequently, they reported quinoxaline-2-carboxylate 1,4-di-*N*-oxide derivatives with 6,7-dimethyl groups that showed low activity. These results confirmed that the chlorine, methyl, or methoxy groups at 7-position reduce the MIC and IC_50_ values. Additionally, they indicated that antibacterial activity depends on the substituent at 2-position, in the following order: benzyl > ethyl > 2-methoxyethyl > allyl > tertbutyl. Interestingly, the best selectivity index (SI) values were obtained from unsubstituted or single substituent compounds at 7-position [14] (Figure 1). 

In 2008, Vicente et al. (2008) indicated that quinoxaline-2-carboxylate derivatives with a benzyl group in the carboxylate enhanced antibacterial activity [15]. Interestingly, they determined that the substituents at 6- and 7-position do not affect antibacterial activity, but they do influence cytotoxicity. In the same year, Villar et al. (2008) found that compound 5, a quinoxaline 1,4-di-*N*-oxide derivative, exhibited in vitro antituberculosis activity comparable to clinically used drugs, although they required a high dose of compound 5 to achieve an equivalent reduction in Colony Forming Units (CFU) in the lung [16]. These results showed the key importance of the chlorine atom at 7-position on the quinoxaline ring. Subsequently, Ancizu et al. (2010) determined in 3-methylquinoxaline-2-carboxamide 1,4-di-*N*-oxide derivatives that the introduction of electron-acceptor substituents on the quinoxaline ring increases the antibacterial activity, although the incorporation of an electron-donor group reduced the activity [17].

New approaches in the development of quinoxalines have been made by other research groups. Das et al. proposed the synthesis of new molecules with an unsaturated conjugated keto group at 2-position (R_2_, Figure 1), which could interact with cellular thiols, affecting mitochondrial functions of rat liver mitochondria and other biochemical processes in bacteria. Interestingly, these kinds of compounds stimulated respiration in rat liver mitochondria, which is indicative of the fact that these compounds exert their mechanism of action through a different pathway than antibacterial drugs, suggesting the possibility of using these kinds of compounds in drug-resistant strains [18]. Also, the fusion between isoniazid and the quinoxaline 1,4-di-*N*-oxide ring has been considered, to obtain molecules that can be orally bioavailable. The biological activity of these kinds of compounds suggested that they may present a dual mechanism of action [19]. In other quinoxaline derivatives, the results of biological activity indicate that the activity significantly depends on the presence of electronegative groups [20]. 

Previous research reports show that quinoxaline 1,4-di-*N*-oxide derivatives are a good option to develop anti-*Mycobacterium tuberculosis* agents. However, to our knowledge, the biological activity of quinoxaline 1,4-di-*N*-oxide derivatives has not been explored against nocardiasis, an infection caused by a bacterium phylogenetically related to *M. tuberculosis*. Therefore, in this study, esters of quinoxaline-7-carboxylate 1,4-di-*N*-oxide were screened in vitro as potential new antinocardial agents and their structure–activity relationship (SAR) was discussed.

## 2. Results

Thirteen compounds derived from methyl, ethyl, isopropyl, and n-propyl quinoxaline-7-carboxylate 1,4-di-*N*-oxide were obtained (Table 1), which presented the analyses of IR, ^1^H-NMR, and UPLC-MS previously reported (see Supplementary Materials).

### 2.1. Antinocardial Activity

The activity values (MIC) of thirteen esters of quinoxaline 1,4-di-*N*-oxide and nine reference drugs against the European reference strain of *N. brasiliensis* CECT3052 and 6 Mexican clinical isolates are shown in Table 1. 

Nine quinoxaline 1,4-di-*N*-oxide derivatives (four methyl, one ethyl, two isopropyl, and two n-propyl esters) showed MICs ≤1 µg/mL against reference strains CECT3052. The best compound was **N-09**, an ethyl ester derivative with a value of MIC < 0.06 µg/mL. This result is better that the ones displayed by the six reference drugs (trimethoprim/sulfamethoxazole, amikacin, amoxicillin, linezolid, and tobramycin). 

The analysis of all compounds evaluated against the clinical isolates (N-100 to N-700) showed that three quinoxaline 1,4-di-*N*-oxide derivatives (**N-05**, **N-09**, and **N-13**) have the best activity against all the strains analyzed, including the reference strain, with MICs values ≤ 1 μg/mL.

In general, these quinoxalines have better antinocardial activity in comparison with most of the reference drugs studied; most of them are in current use for nocardiosis treatment, so the potential use of these compounds deserves further preclinical studies.

Compound **N-09** was the best antinocardial agent, which had a thienyl, trifluoromethyl, and ethyl groups at R1-, R2-, and R3-position, respectively, on the quinoxaline 1,4-di-*N*-oxide ring. Additionally, the biological behavior (SAR) of quinoxaline 1,4-di-*N*-oxide derivatives against clinical isolates was the same as in the reference strain. To our knowledge, this is the first report of quinoxaline 1,4-di-*N*-oxide derivatives with antinocardial activity. The results of the antinocardial activity of reference drugs show that the strain most susceptible to all the drugs analyzed was the CECT-3052 strain, and the drugs with the lowest activity were trimethoprim/sulfamethoxazole, amikacin, and tobramycin (Table 1).

### 2.2. Cytotoxic Activity

Additionally, the cytotoxic activity was determined for the thirteen esters of quinoxaline 1,4-di-*N*-oxide derivatives at three concentrations (100, 10, and 0.5 µg/mL) against macrophages J774A, and the half-maximal cytotoxic concentration (CC_50_) was calculated. Table 1 summarizes the results of the cytotoxicity analysis. A variability in the cytotoxicity of the compounds was observed: five compounds (**N-01**, **N-02**, **N-03**, **N-08**, and **N-10**) showed a very low toxicity, four showed intermediate toxicity, and four more compounds were very toxic.

## 3. Discussion

### 3.1. Structure–Activity Relationship

Among the different substituents present at R_1_ and R_2_, a clear tendency can be noted for the presence of the trifluoromethyl group at R_2_ as a substitution favoring antinocardial activity. Of the nine compounds that showed an MIC ≤ 1 µg/mL against the reference strain CECT3052, seven derivatives bear a trifluoromethyl group, while the remaining two bear a methyl group. This tendency suggests the benefit of the electron-withdrawing substituents permitting an increased activity. This is further emphasized when comparing **N-05** to **N-03**, where the change from methyl to trifluoromethyl at R_2_ permits a 25-fold increase in the activity. The most active compound against the reference strain **N-09** bears a thienyl group at R_1_. This substitution at R_1_ is present in three other derivatives with MIC values of 0.25 µg/mL (**N-06**, **N-11**, and **N-13**), further emphasizing the benefit of this ring at R_1_. Comparing the R_3_ position, it may be observed that only the presence of an ethyl ester at R_3_ in **N-09** permitted a 4-fold increase in activity in comparison to methyl (**N-06**), n-propyl (**N-11**), and isopropyl (**N-13**) esters, suggesting that a mid-size ester permits a better activity over a small ester and bulkier esters. An additional comparison can be made for the ester at R_3_ position comparing **N-12** to **N-04** where the derivative with the isopropyl is 2-fold better than methyl substituted, yet the comparison of **N-01** to **N-10** shows a 2-fold better activity for methyl over n-propyl ester at R_3_, thus suggesting that the main contributing factors are at R_1_ and R_2_.

For the clinical isolates N100-N700, the tendency observed was consistent with the observed activity against the reference strain Nocardia. Three derivatives (**N-05**, **N-09**, and **N-13**) had MIC values ≤ 1 µg/mL against all isolates, two of which bear a thienyl at R_1_ and the last bears a phenyl ring; all three bear a trifluoromethyl at R_2_. Derivative **N-11** has an MIC value ≤ 1 µg/mL against five isolates, while **N-08** has an MIC value ≤ 1 µg/mL against four isolates; both bear a trifluoromethyl at R_2_, with a thienyl and an acetyl at R_1_ for **N-11** and **N-08** respectively. Altogether these data support similar conclusions as those drawn from the reference strain.

The general trend observed with quinoxaline-1,4-di-*N*-oxide esters is an increase in activity in the presence of electron-withdrawing substituents, such as trifluoromethyl. This pattern is consistent with reports on antimycobacterial agents [21], a class of organisms related to Nocardia. The literature suggests that quinoxaline-1,4-di-*N*-oxide exerts its antibacterial effects by causing DNA damage upon bioreduction [22]. This bioreduction appears to be more efficient when electron-withdrawing groups are present, as they facilitate the reduction process. As a result, reactive oxygen species (ROS) are generated, which may contribute to the compound’s antinocardial activity. Therefore, the mode of action might be linked to the DNA damage induced by ROS generated during bioreduction.

In comparison to the activity observed for these compounds as antimycobacterial agents, a closely related organism shows that these exert their activity at a similar concentration range. These compounds, reported by our research group in 2021, range in MIC values from 0.08 to 3.47 µg/mL; a chart presenting a one-to-one comparison may be consulted in the Supplementary Materials. This suggests that they may share a similar mode of action for both organisms, though it is still unknown.

### 3.2. Selectivity of Antinocardial Activity

Five quinoxaline derivatives had CC_50_ values over 170 µg/mL; four out of these five derivatives bear a methyl group at R_2_ (**N-01**, **N-02**, **N-03**, and **N-10**), while the last one bears a trifluoromethyl (**N-08**). These data suggest that though the trifluoromethyl group is beneficial to increase the antinocardial activity, it also leads to an unspecific cytotoxicity. The comparison of **N-08** to **N-09**, where the only change is the substitution of a methyl for a thienyl, shows that this modification results in a 10-fold cytotoxicity increase. Still, the concentration at which the derivatives exert their activity permits a certain level of selectivity as antinocardial agents instead of them behaving as cytotoxic molecules. Considering the Nocardia reference strain, eight out of the thirteen derivatives have a selective index over 100-fold more active against Nocardia over macrophages. The five most selective derivatives are **N-01**, **N-09**, **N-10**, **N-12**, and **N-13** with SI values of 720, 456, 300, 201, and 200, respectively. Two sets of substituents are present among these five derivatives: methoxy at R_1_ and methyl at R_2_ for **N-01** and **N-10**, and thienyl at R_1_ and trifluoromethyl at R_2_ for **N-09** and **N-13**.

**Figure 2:**
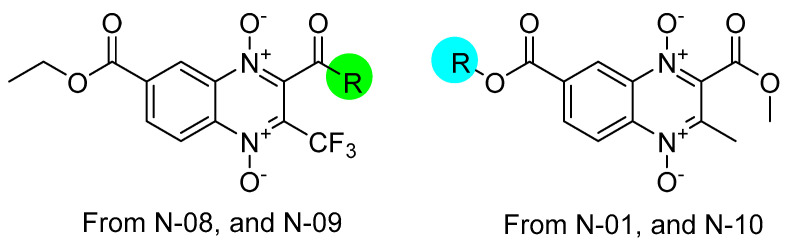
Based on the observed selectivity index, two proposed substructures of quinoxaline 1,4-di-*N*-oxide for further optimization at the colored green and blue areas.

Considering the six clinical isolates, the three compounds with the most selectivity throughout are **N-08** (SI values ranging from 129 to 519), **N-09** (SI values ranging from 114 to 459), and **N-01** (SI values ranging from 180 to 720). The highest selectivity index observed was for **N-10** against isolate N300 (SI 1203), yet this level of selectivity was not maintained throughout all isolates. It may be observed that two of these most selective compounds (**N-08** and **N-09**) bear a trifluoromethyl at R_2_ and an ethyl ester at R_3_, while **N-01** and **N-10** bear a methyl ester at R_1_ and a methyl at R_2_, suggesting the substructures shown in Figure 2 as potential optimizing starting points.

## 4. Materials and Methods

### 4.1. Quinoxaline 1,4-di-N-oxide Derivatives

**Figure 3:**
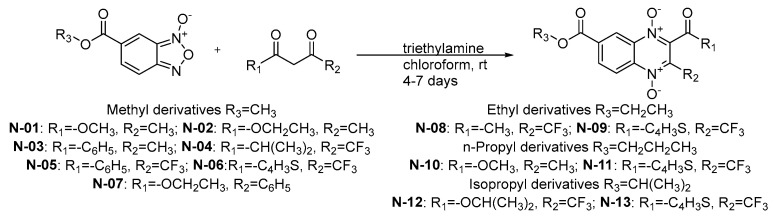
Synthetic scheme for quinoxaline-1,4-di-*N*-oxide derivatives **N-01** to **N-13** [23].

From a series of compounds previously reported by Palos et al. (2018), 13 compounds derived from methyl, ethyl, isopropyl, and n-propyl esters of quinoxaline 1,4-di-*N*-oxide were selected because they had activity against *M. tuberculosis* [8]. All reagents were purchased from chemical vendor Sigma-Aldrich (Toluca, Mexico). The compounds were obtained following the procedure (Figure 3) previously reported by Gomez-Caro et al. (2011) [23]. The corresponding β-diketone (4 mmol) was added to the solution of the appropriate benzofuroxan-*N*-oxide (2 mmol) in dry chloroform (15 mL) while on an ice bath. Triethylamine (TEA) was added (1 mL), and the reaction mixture was stirred at room temperature for 4–7 days. The reaction mixture was concentrated under reduced pressure and purified by column chromatography. The purified products were analyzed by IR, 1H-NMR, and UPLC-MS, which have been previously reported [8,24,25,26,27]. Additionally, nine antibiotics were used as reference drugs (trimethoprim/sulfamethoxazole, amikacin, amoxicillin, ciprofloxacin, clarithromycin, doxycycline, linezolid, rifampin, and tobramycin).

### 4.2. Biological Material

The European reference strain CECT3052 from *N. brasiliensis* and 6 clinical isolates from Mexican patients were used. The clinical isolates were recovered from mycetoma cases in conventional media and identified by the VITEK-MS identification system (bioMérieux Inc., Grenoble, France).

### 4.3. Biological Evaluation In Vitro against N. brasiliensis

For the evaluation of the effect of the quinoxaline 1,4-di-*N*-oxide derivatives against *N. brasiliensis*, a microplate flurometric assay was adapted according to CLSI guidelines, using the Alamar blue dye as viability indicator [28] (2012). The quinoxaline 1,4-di-*N*-oxide derivatives were prepared in dimethyl sulfoxide (DMSO) under sterile conditions at a concentration of 1 mg/mL, stored at −70 °C until use. The assay was performed on a 96-well culture microplate. The in vitro test was performed by analyzing six concentrations of the compounds in a range of serial dilutions with a maximum concentration of 50 µg/mL; the Muller–Hinton broth cation-adjusted medium (BBL, Becton-Dickinson) was used. The *N. brasiliensis* reference strain or clinical isolates were initially grown in trypticase soy agar medium (BBL, Becton-Dickinson) during 4 days at 37 °C, one or two colonies were taken to obtain a fine bacterial suspension that was adjusted to McFarland nephelometer standard number 0.5, and the big clumps were eliminated by sedimentation. Once the compounds were diluted in the 96-well plate, 100 µL of the bacterial-adjusted suspension was placed in each well and incubated at 37 °C during 72 h, and then 20 µL of the Alamar blue dye solution (Thermo Fisher Scientific) was added to each well, leaving the plates for incubation during an additional 24 h. The fluorometric readings were obtained in a plate fluorometer (Fluoroskan Ascent FL, Thermo Fisher Scientific Corporation), at 544 nm excitation and 590 nm emission.

### 4.4. Cytotoxic Activity and Selectivity Index Determination

The test was carried out on macrophage monolayers of the cell line J774A.1 (ATCC TIB-67), prepared in 96-well plates with 10,000 cells/well in the F-12 culture medium added with 10% fetal calf serum; three different concentrations of the compounds were used, from 100 to 0.5 µg/mL. Cell viability was analyzed at 6 h, with Alamar Blue solution following a cytotoxicity test [28]. The cytotoxicity of the compounds **N-01**, **N-05**, **N-08**, **N-09**, **N-10**, **N-11**, **N-12**, and **N-13** was determined. Briefly, cell monolayers were prepared in 96-well plates with 10,000 cells per well in Ham’s F-12 medium supplemented with 10% fetal bovine serum (FBS, By products, Guadalajara, Mexico) and antibiotics (penicillin and gentamicin). Three concentrations, from 100 to 0.5 µg/mL of each of the compounds were tested by triplicate. The cells were incubated for a period of 6 h. Before completing this period, 20 µL of Alamar blue solution was added to each well, quantifying the relative fluorescence units with a fluorometer (Fluoroskan Ascent FL, Thermo Fisher Scientific, Vantaa, Finland). The percentage of cytotoxicity at each concentration was determined by comparing the test values against the control of cells without treatment. The CC_50_ was determined with a linear regression analysis (Sigma Plot v12, Palo Alto, California, USA). The SI was calculated as the ratio of the CC_50_ on the macrophage cell line J774A.1 and the MIC value against *N. brasiliensis* and clinical isolates.

## 5. Conclusions

In this study, nine compounds evaluated (**N-01**, **N-04**, **N-05**, **N-06**, **N-09**, **N-10**, **N-11**, **N-12**, and **N-13**) had good activity against the reference strain *N. brasiliensis* CECT-3052 (MIC < 1 µg/mL). Furthermore, four compounds (**N-05**, **N-09**, **N-11**, and **N-13**) showed activity against six clinical isolates evaluated, with MIC values < 1 µg/mL. Additionally, ciprofloxacin and rifampin showed the best antinocardial activity against the reference strain and amikacin against the clinical isolates. Finally, the cytotoxic evaluation showed that five compounds (**N-01**, **N-02**, **N-03**, **N-08**, and **N-10**) had low toxic activity. Therefore, the compounds with low cytotoxic and high antinocardial effect were **N-01**, **N-08**, and **N-10**. These results highlight the importance of quinoxaline 1,4-di-*N*-oxide as a scaffold to develop new and potent antinocardial agents.

## Figures and Tables

**Table 1:** Minimum Inhibitory Concentration (µg/mL) of esters of quinoxaline 1,4-di-*N*-oxide derivatives and nine reference drugs against *N. brasiliensis,* six clinical isolates (N-100 to N-700), cytotoxic activity against macrophage J774A.1, and selectivity index.

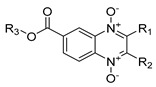																		
Code	R_1_	R_2_	R_3_	J774A.1 *	CECT3052	SI	N100	SI	N200	SI	N300	SI	N400	SI	N600	SI	N700	SI
**N-01**	COOCH_3_	CH_3_	CH_3_	360 ± 4.5	0.5	720	0.5	720	2	180	3.12	115	1.56	231	6.25	57	1.56	231
**N-02**	COOCH_2_CH_3_	CH_3_	CH_3_	214 ± 1	ND	ND	3.12	68	6.25	34	25	8	6.25	34	50	4	6.25	34
**N-03**	COC_6_H_5_	CH_3_	CH_3_	179 ± 3.8	6.25	28	6.25	28	50	3	25	7	25	7	25	7	12.5	14
**N-04**	COCH(CH_3_)_2_	CF_3_	CH_3_	36 ± 5.9	0.5	71	0.5	71	1.56	22	1.56	22	1.56	22	2	17	2	17
**N-05**	COC_6_H_5_	CF_3_	CH_3_	47 ± 4.1	0.25	188	0.25	188	0.5	94	0.5	94	1	47	0.5	94	0.5	94
**N-06**	COC_4_H_3_S	CF_3_	CH_3_	36 ± 7.2	0.25	142	0.25	142	2	17	2	17	1.56	22	0.5	71	1	35
**N-07**	COOCH_2_CH_3_	C_6_H_5_	CH_3_	75 ± 12.6	<1.56	48	6.25	12	12.5	6	6.25	12	3.12	24	12.5	6	3.12	24
**N-08**	COCH_3_	CF_3_	CH_3_CH_2_	260 ± 2	ND	ND	0.5	519	0.5	519	1	259	2	129	1	259	2	129
**N-09**	COC_4_H_3_S	CF_3_	CH_3_CH_2_	28 ± 3.5	<0.06	46	0.06	459	0.25	114	0.25	114	0.25	114	0.25	114	0.12	237
**N-10**	COOCH_3_	CH_3_	CH_3_CH_2_CH_2_	301 ± 4.4	1	300	1	300	3.12	96	0.25	1203	3.12	96	3.12	96	3.12	96
**N-11**	COC_4_H_3_S	CF_3_	CH_3_CH_2_CH_2_	41 ± 6.5	0.25	164	0.25	164	0.5	82	0.5	82	0.5	82	0.25	164	2	20
**N-12**	COCH(CH_3_)_2_	CF_3_	(CH_3_)_2_CH	50 ± 6.1	0.25	201	0.25	201	1.56	32	1	50	2	25	1.56	32	2	25
**N-13**	COC_4_H_3_S	CF_3_	(CH_3_)_2_CH	50 ± 3.5	0.25	200	0.25	200	0.5	100	0.25	200	0.25	200	1	50	0.5	100
TR1/SUL					0.25/4.75		0.5/9.5		0.25/4.75		1/19		0.25/4.75		0.25/4.75		0.25/4.75	
AMI					0.12		0.25		0.5		0.25		0.25		0.5		0.25	
AMO					2		64		4		2		32		4		8	
CIP					1		2		2		2		2		1		1	
CLA					1		16		2		1		8		2		4	
DOX					1		1		0.25		1		1		0.5		1	
LIN					2		1		1		1		1		1		1	
RIF					8		32		0.25		4		0.25		0.25		64	
TOB					0.12		0.25		0.25		0.25		0.25		0.25		0.25	

Trimethoprim/sulfamethoxazole (TRI/SUL), amikacin (AMI), amoxicillin (AMO), ciprofloxacin (CIP), clarithromycin (CLA), doxycycline (DOX), linezolid (LIN), rifampin (RIF), tobramycin (TOB), and selectivity index (SI). * Values of half-maximal cytotoxic concentration (µg/mL) ± standard deviation.

## Data Availability

The authors may be contacted regarding data.

## Supplementary Materials

The following supporting information can be downloaded at: https://www.mdpi.com/article/10.3390/molecules29194652/s1. Structural characterization of compounds (**N-01** to **N-013**).
