# Supplementary material for: Quinoxaline 1,4-di-N-oxide Derivatives as New Antinocardial Agents
Source: Molecules. 2024 Sep 30;29(19):4652. doi: 10.3390/molecules29194652 (PMC11478375; doi:10.3390/molecules29194652)
Supplement: Supplementary file 1 [file molecules-29-04652-s001.zip › molecules-3136837-supplementary.pdf]

SUPPLEMENTARY INFORMATION

Quinoxaline 1,4-di-*N*-oxide as New Antinocardia Agents

Isidro Palos[1], Alonzo Gonzalez-Gonzalez[2], Alma D. Paz-González[2], Debasish Bandyopadhyay[3,4], Marlene S. Galeana-Salazar[1,5], Norma Paniagua-Castro[6], Jorge Ismael Castañeda-Sánchez[5], Julieta Luna-Herrera[7]*, Gildardo Rivera[2]*

1 Unidad Académica Multidisciplinaria Reynosa-Rodhe, Universidad Autónoma de Tamaulipas, 88779 Reynosa, México.
2 [2]Laboratorio de Biotecnología Farmacéutica, Centro de Biotecnología Genómica, Instituto Politécnico Nacional, Boulevard del Maestro, s/n, Esq. Elías Piña, Reynosa 88710, México.
3 Department of Chemistry, The University of Texas Rio Grande Valley, 1201 West University Drive, Edinburg, Texas 78539, USA.
4 School of Earth Environment & Marine Sciences (SEEMS), The University of Texas Rio Grande Valley, 1201 West University Drive, Edinburg, Texas 78539, USA
5 Departamento de Fisiología, Escuela Nacional de Ciencias Biológicas, Instituto Politécnico Nacional, Ciudad de México 11340, México.
6 Departamento de Sistemas Biológicos, Universidad Autónoma Metropolitana-Xochimilco, Ciudad de México 09460, México.
7 Departamento de Inmunología, Escuela Nacional de Ciencias Biológicas, Instituto Politécnico Nacional, Ciudad de México 11340, México.

*Corresponding author: Gildardo Rivera, Laboratorio de Biotecnología Farmacéutica, Centro de Biotecnología Genómica, Instituto Politécnico Nacional, Boulevard del Maestro, s/n, Esq. Elías Piña, Col. Narciso Mendoza, 88710, Reynosa, Tamaulipas, México giriveras@ipn.mx

Structural elucidation

N-01: 3,6-bis(methoxycarbonyl)-2-methylquinoxaline 1,4-dioxide. This compound was obtained in 12% yield to be a light yellow solid (m.p. 117-118 °C). IR (KBr): 1715.30 (C=O), 1333.57 (N-oxide) cm^{-1}. ^1H NMR (400 MHz, DMSO-d6) ppm: 2.44 (s, 3H, CH_3), 3.97 (s, 3H, $COOCH_3$), 4.03 (s, 3H, CH_3OOC), 8.38 (d, J = 8.93 Hz, 1H, H5), 8.56 (d, J = 8.89 Hz, 1H, H6), 8.85 (s, 1H, H8).

N-02: 3-(ethoxycarbonyl)-6-(methoxycarbonyl)-2-methylquinoxaline 1,4-dioxide. This compound was obtained in 17% yield to be a orange-yellow solid (m.p. 108-109 °C). IR (KBr): 1719.20 and 1741.12 (C=O), 1328.02 (N-oxide) cm^{-1}. ^1H NMR (400 MHz, DMSO-d6) ppm: 1.36 (t, 3H, $COOCH_2CH_3$), 2.45 (s, 3H, CH_3), 3.97 (s, 3H, CH_3OOC), 4.5 (q, J1 = 7.10 Hz, J2 = 7.12 Hz, and J3 = 7.10 Hz, 2H, $COOCH_2CH_3$), 8.39 (d, J = 8.98 Hz, 1H, H5), 8.56 (d, J = 8.99 Hz, 1H, H6), 8.85 (s, 1H, H8).

N-03: 3-benzoyl-6-(methoxycarbonyl)-2-methylquinoxaline 1,4-dioxide. This compound was obtained in 10.9% yield to be a pale yellow solid (m.p 124 °C). IR (KBr): 2949 (ArC-H), 1726 and 1674 (C=O), 1328 (N-oxide) cm^{-1}. ^1H NMR (400 MHz, DMSO-d6) ppm: 2.54 (s, 3H, CH_3), 4.04 (s, 3H, CH_3OOC), 7.56 (t, J = 7.75 Hz, 2H, H3 and H5, C_6H_5), 7.72 (t, J = 8.08 Hz, 1H, H4, C_6H_5), 7.90–7.93 (m, 2H, H2 and H6, C_6H_5), 8.52 (d, J = 9.0 Hz, 1H, H5), 8.76 (d, J = 9.01 Hz, 1H, H6), 9.22 (s, 1H, H8).

N-04: 3-isobutyryl-6-(methoxycarbonyl)-2-(trifluoromethyl) quinoxaline 1,4-dioxide. This compound was obtained in 23.9% yield to be a light yellow solid (m.p. 123-124 °C). IR (KBr): 2923 (ArC–H), 1730 (C=O), 1356 (N-oxide), 1293 and 1187 (Ar-CF_3) cm^{-1}. ^1H NMR (400 MHz, DMSO-d6) ppm: 1.27 (s, 6H, $CH(CH_3)_2$), 3.17 (q, J1 = 14.09 Hz, J2 = 7.05 Hz, 1H, $CH(CH_3)_2$), 4.07 (s, 3H, CH_3OOC), 8.57 (d, J = 8.98 Hz, 1H, H5), 8.64 (d, J = 8.95 Hz, 1H, H6), 9.27 (s, 1H, H8).

N-05: 3-benzoyl-6-(methoxycarbonyl)-2-(trifluoromethyl) quinoxaline 1,4-dioxide. This compound was obtained in 10.9% yield to be a yellow solid (m.p. 132 °C). IR (KBr): 2957 (ArC–H), 1732 and 1689 (C=O), 1337 (N-oxide), 1255 and 1164 (Ar–CF_3) cm^{-1}. ^1H NMR (400 MHz, DMSO-d6) ppm: 4.01 (s, 3H, CH_3OOC), 7.61 (t, J = 7.09 Hz, 2H, H3 and H5, C_6H_5), 7.79 (t, J = 7.24 Hz, 1H, H4, C_6H_5), 8.15 (d, J = 7.82 Hz, 2H, H2 and H6, C_6H_5), 8.52–8.54 (m, 2H, H5 and H6), 8.97 (s, 1H, H8).

N-06: 6-(methoxycarbonyl)-3-(thiophene-2-carbonyl)-2-(trifluoromethyl) quinoxaline 1,4-dioxide. This compound was obtained in 5.6% yield to be a pale yellow solid (m.p. 133 °C). IR (KBr): 2955 (ArC–H), 1732 and 1664 (C=O), 1360 (N-oxide), 1264 and 1162 (Ar–CF_3) cm^{-1}. ^1H NMR (400 MHz, DMSO-d6) ppm: 4.01 (s, CH_3OOC), 7.22 (t, J = 4.41 Hz, 1H, H4, C_4H_3S), 7.63 (d, J = 3.77 Hz, H5, C_4H_3S), 7.9 (d, J = 4.85 Hz, H3, C_4H_3S), 8.58 (d, J = 8.86 Hz, 1H, H5), 8.67 (d, J = 8.89 Hz, 1H, H6), 9.31 (s, 1H, H8).

N-07: 3-(ethoxycarbonyl)-6-(methoxycarbonyl)-2-phenylquinoxaline 1,4-dioxide. This compound was obtained in 17% yield to be an orange-yellow solid (m.p. 126-127 °C). IR (KBr): 1715.30 and 1726.88 (C=O), 1327.65 (N-oxide) cm^{-1}. ^1H NMR (400 MHz, DMSO-d6) ppm: 1.40 (t, 3H, $COOCH_2CH_3$), 4.16 (s, 3H, CH_3OOC), 4.45 (q, J1 = 6.67 Hz, J2 = 6.40 Hz, 2H, $COOCH_2CH_3$), 7.57 (s, 5H, C_6H_5), 8.44 (d, J = 8.59 Hz, 1H, H5), 8.61 (t, J = 7.98 Hz, 1H, H6), 8.95 (d, J = 8.99 Hz, 1H, H8).

N-08: 3-acetyl-6-(ethoxycarbonyl)-2-(trifluoromethyl) quinoxaline 1,4-dioxide. This compound was obtained in 11.9% yield to be a light yellow solid (m.p. 124 °C). IR (KBr): 2978 (ArC–H), 1746 (C=O), 1355 (N-oxide), 1271 and 1158 (Ar–CF_3) cm^{-1}. ^1H NMR (400 MHz, DMSOd6) ppm: 1.40 (t, J = 7.11 Hz, 3H, CH_3CH_2OOC), 2.62 (s, 3H, $COCH_3$), 4.45 (q, J1 = 7.12 Hz, J2 = 7.13 Hz, 2H, CH_3CH_2OOC), 8.52 (d, J = 8.95 Hz, 1H, H5), 8.58 (d, J = 8.96 Hz, 1H, H6), 8.92 (s, 1H, H8).

N-09: 6-(ethoxycarbonyl)-3-(thiophene-2-carbonyl)-2-(trifluoromethyl) quinoxaline 1,4-dioxide. This compound was obtained in 23.2% yield to be a light yellow solid (m.p. 135 °C). IR (KBr): 2987 (ArC–H), 1726 and 1665 (C=O), 1336 (N-oxide), 1286 and 1161 (Ar–CF_3) cm^{-1}. ^1H NMR (400 MHz, DMSO-d6) ppm:

1.41 (t, J = 7.1 Hz, 3H, CH_3CH_2OOC), 4.47 (q, J1 = 7.07 Hz, J2 = 7.12 Hz, 2H, CH_3CH_2OOC), 7.32 (d, J = 4.7 Hz, 1H, H4, C_4H_3S), 8.24 (d, J = 4.59 Hz, H5, C_4H_3S), 8.3 (d, J = 4.8 Hz, H3, C_4H_3S), 8.51 (d, J = 8.95 Hz, 1H, H5), 8.56 (d, J = 8.96 Hz, 1H, H6), 8.96 (s, 1H, H8).

N-10: 3-(methoxycarbonyl)-2-methyl-6-(propoxycarbonyl) quinoxaline 1,4-dioxide. This compound was obtained in 11.85% yield to be a yellow solid (m.p. 129 °C). IR (KBr): 2964.93 (ArC-H), 1745.58 (C=O), 1331 (N-oxide) cm^{-1}. ^{1}H-NMR (400 MHz, DMSO-d6) ppm: 1.0 (t, 3H, CH3(CH2)2O), 1.74–1.84 (m, 2H, CH3(CH2)2O), 2.44 (3H, CH3), 4.03 (s, 3H, COOCH3), 4.32–4.36 (m, 2H, CH3(CH2)2O), 8.35–8.40 (m, 1H, H5), 8.51–8.57 (m, 1H, H6), 8.85–8.90 (m, 1H, H8).

N-11: 6-(propoxycarbonyl)-3-(thiophene-2-carbonyl)-2-(trifluoromethyl) quinoxaline 1,4-dioxide. This compound was obtained in 6.51% yield to be a pale yellow solid (m.p. 139-140 °C). IR (KBr): 2965 (ArC-H), 1722 and 1662 (C=O), 1331 (N-oxide), 1284 and 1165 (Ar-CF3) cm^{-1}. ^{1}H-NMR (400 MHz, DMSO-d6) ppm: 1.0 (t, 3H, CH3(CH2)2O), 1.77–1.86 (m, 2H, CH3(CH2)2O), 4.37–4.40 (m, 3H, CH3(CH2)2O), 7.30–7.34 (m, 1H, C4H3S), 8.25 (m, 1H, C4H3S), 8.31 (m, 1H, C4H3S), 8.5–8.7 (m, 2H, H5, H6), 8.96 (s, 1H, H8).

N-12: 3-isobutyryl-6-(isopropoxycarbonyl)-2-(trifluoromethyl) quinoxaline 1,4-dioxide. This compound was obtained in 16.0% yield to be a light yellow solid (m.p. 135 °C). IR (KBr): 2983 (C-H), 1715 and 1678 (C=O), 1325 (N-oxide), 1283 and 1178 (Ar-CF3) cm^{-1}. ^{1}H-NMR (400 MHz, DMSO-d6) ppm: 1.30 (s, 6H, COCH(CH3)2), 1.33 (s, 6H, (CH3)2CH), 1.4 (d, J1 = 6.25 Hz, 1H, COCH(CH3)2), 5.25 (q, J1 = 6.41 Hz, J2 = 12.54 Hz, (CH3)2-CH-), 8.5 (d, J = 8.94 Hz, 1H, H5), 8.6 (d, J = 8.88 Hz, 1H, H6), 8.88 (s, 1H, H8).

N-13: 6-(isopropoxycarbonyl)-3-(thiophene-2-carbonyl)-2-(trifluoromethyl) quinoxaline 1,4-dioxide. This compound was obtained in 2.5% yield to be a pale yellow solid (m.p. 140-141 °C). IR (KBr): 2968 (C-H), 1721 and 1661 (C=O), 1332 (N-oxide), 1285 and 1153 (Ar-CF3) cm^{-1}. ^{1}H-NMR (400 MHz, DMSO-d6) ppm: 1.40 (m, 6H, (CH3)2CH), 5.24–5.29 (m, (CH3)2-CH-), 7.31 (s, 1H, H4, C4H3S), 8.24 (s, H5, C4H3S), 8.30 (s, H3, C4H3S), 8.51 (s, 1H, H5), 8.54 (s, Hz, 1H, H6), 8.94 (s, 1H, H8).

NMR, and UPLC-MS Spectra

N-01

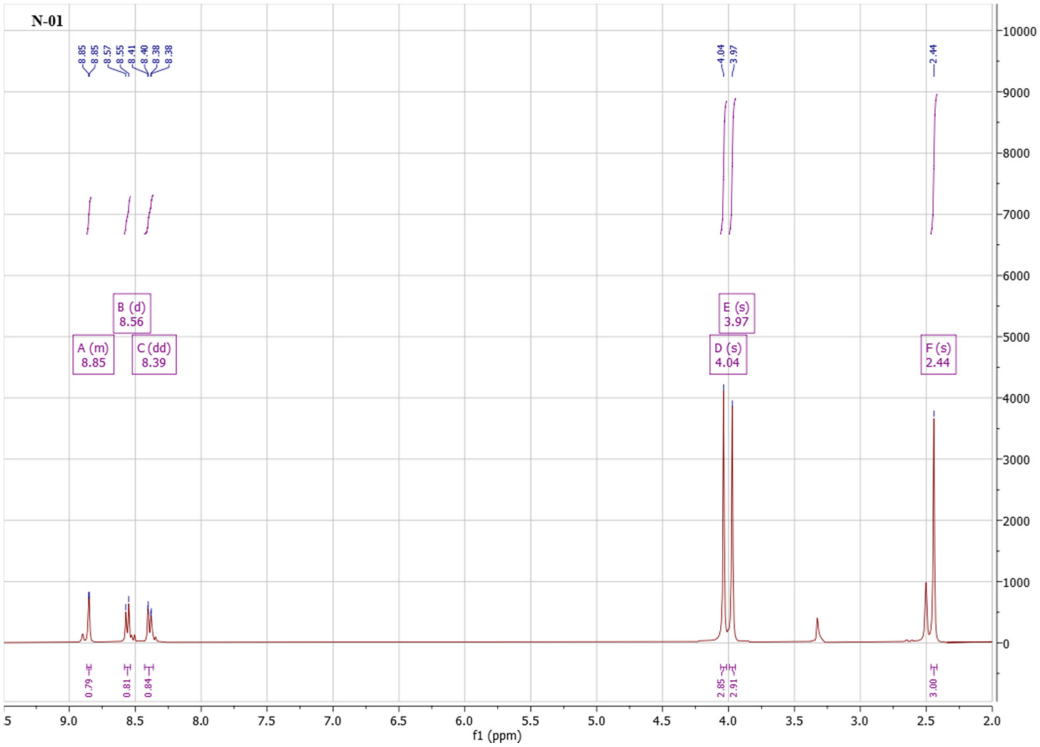

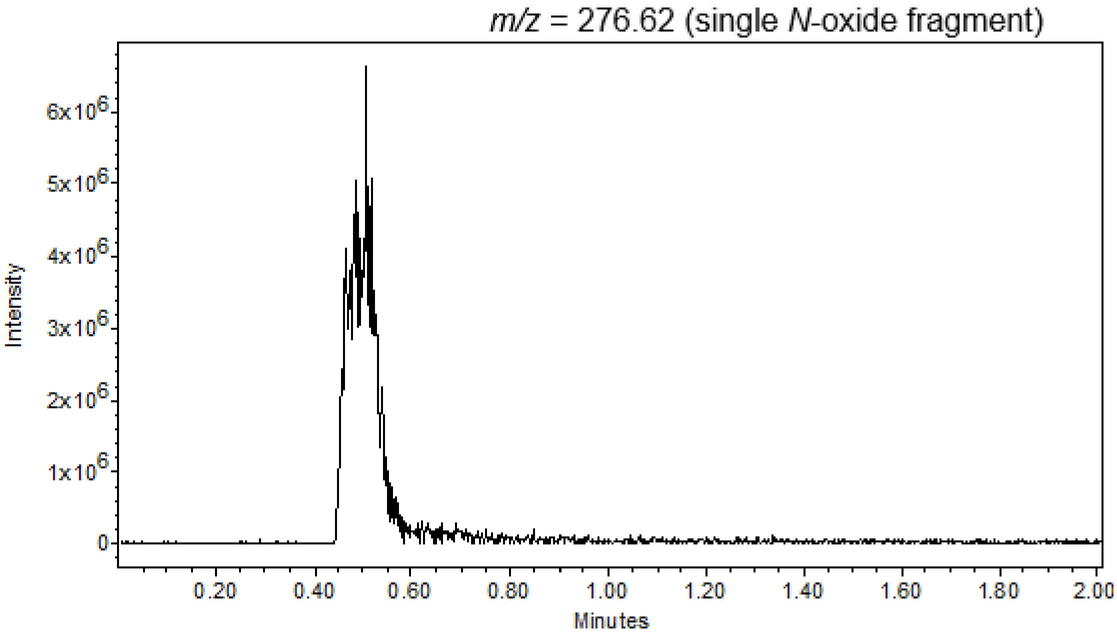

N-02

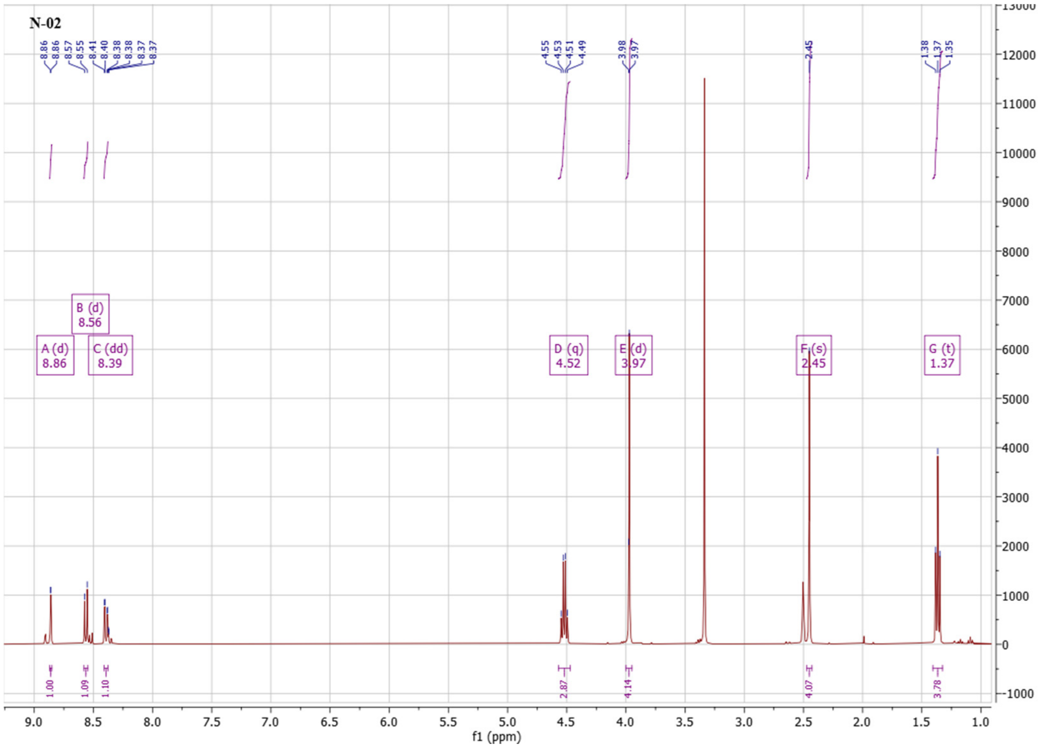
N-02
A (d)
8.86
B (d)
8.56
C (dd)
8.39
D (q)
4.52
E (d)
3.97
F (s)
2.45
G (t)
1.37
f1 (ppm)

N-03

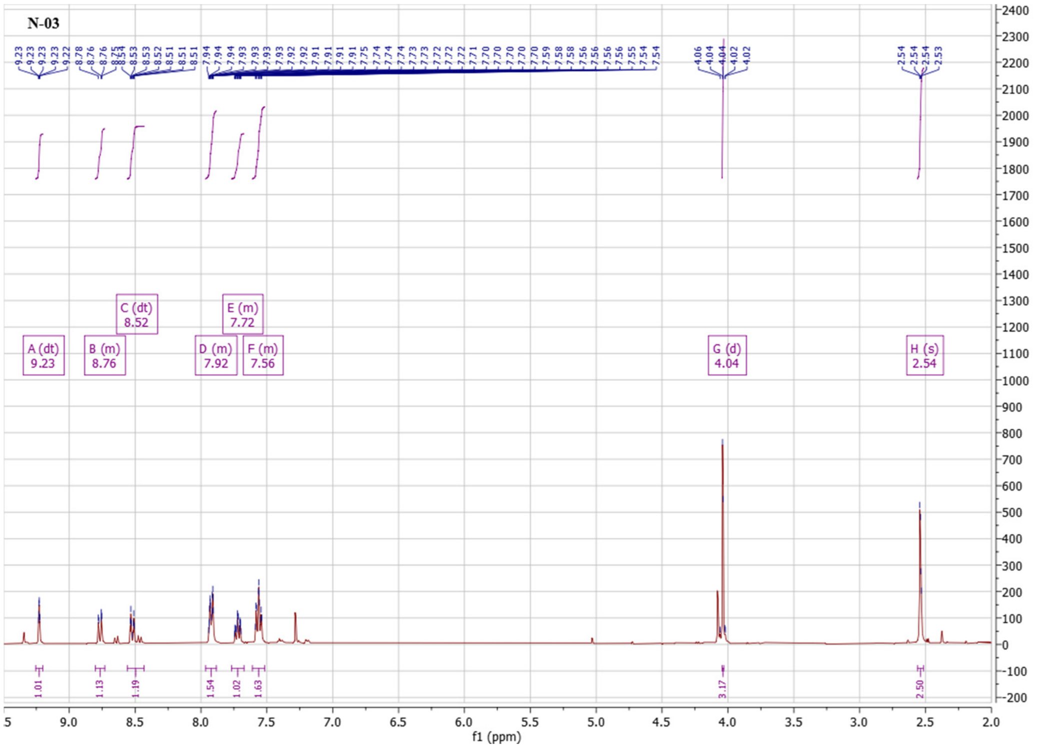
N-03
A (dt)
9.23
B (m)
8.76
C (dt)
8.52
D (m)
7.92
E (m)
7.72
F (m)
7.56
G (d)
4.04
H (s)
2.54
f1 (ppm)

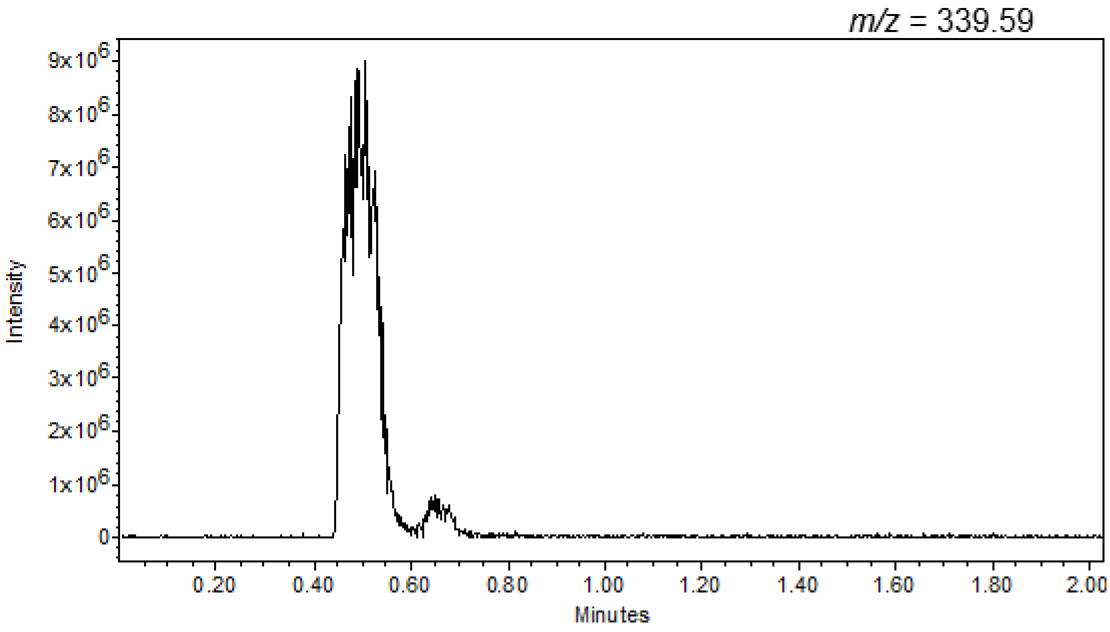
m/z = 339.59
Intensity
Minutes
9x10^6
8x10^6
7x10^6
6x10^6
5x10^6
4x10^6
3x10^6
2x10^6
1x10^6
0
0.20
0.40
0.60
0.80
1.00
1.20
1.40
1.60
1.80
2.00

N-04

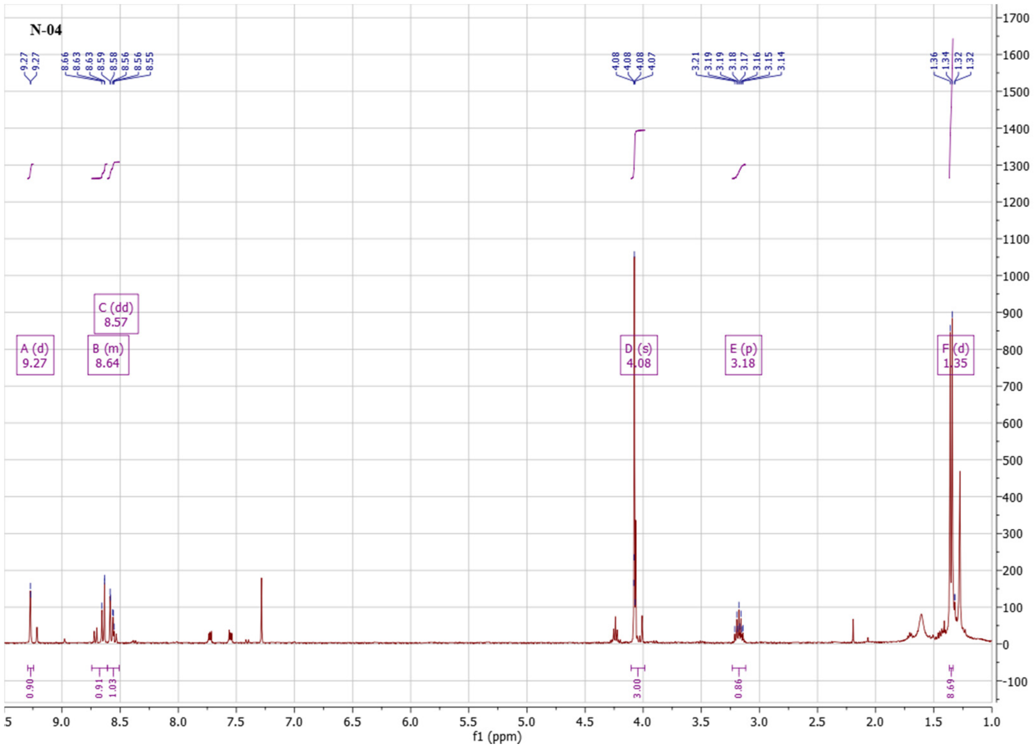
N-04
A (d)
9.27
B (m)
8.64
C (dd)
8.57
D (s)
4.08
E (p)
3.18
F (d)
1.35
f1 (ppm)

N-05

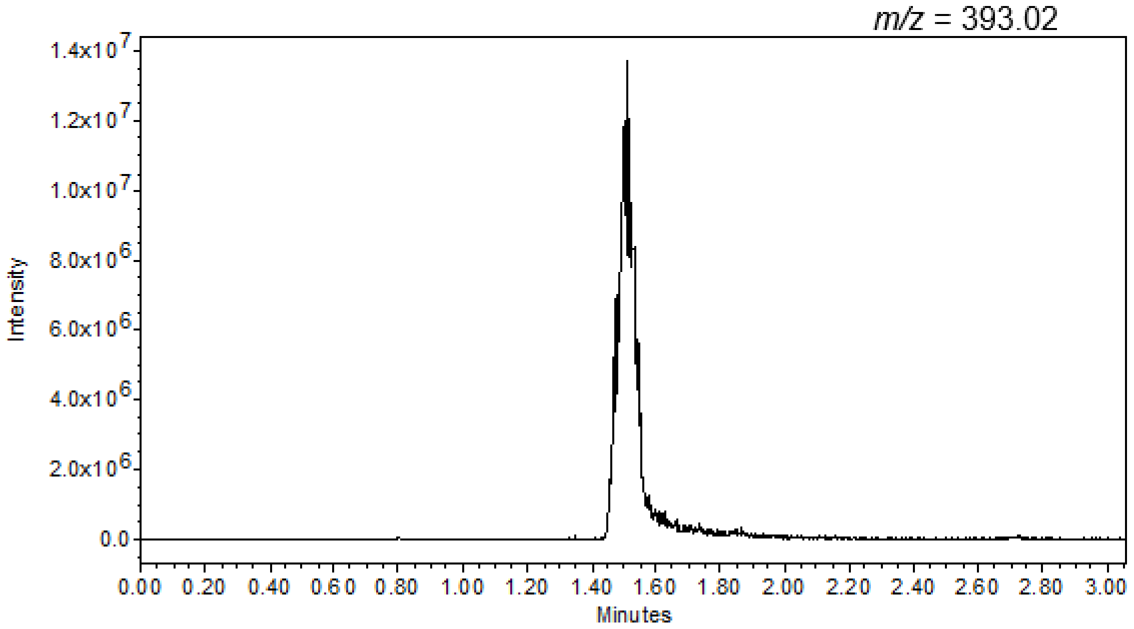
m/z = 393.02
Intensity
Minutes

N-06

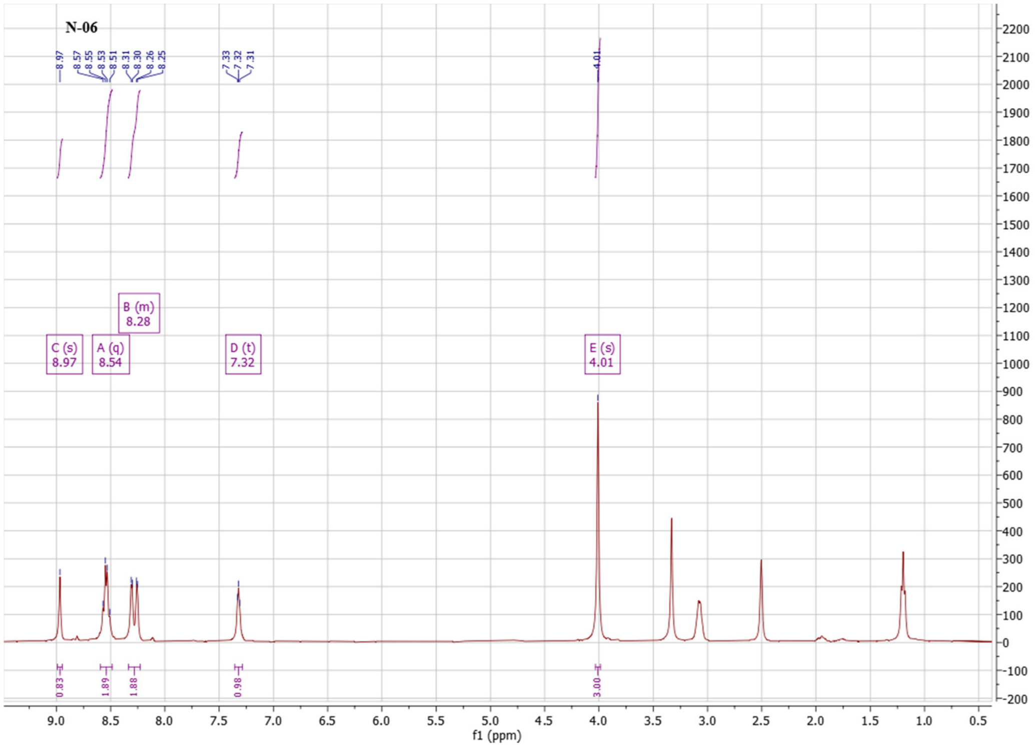
N-06
C (s) 8.97
A (q) 8.54
B (m) 8.28
D (t) 7.32
E (s) 4.01
f1 (ppm)

N-07

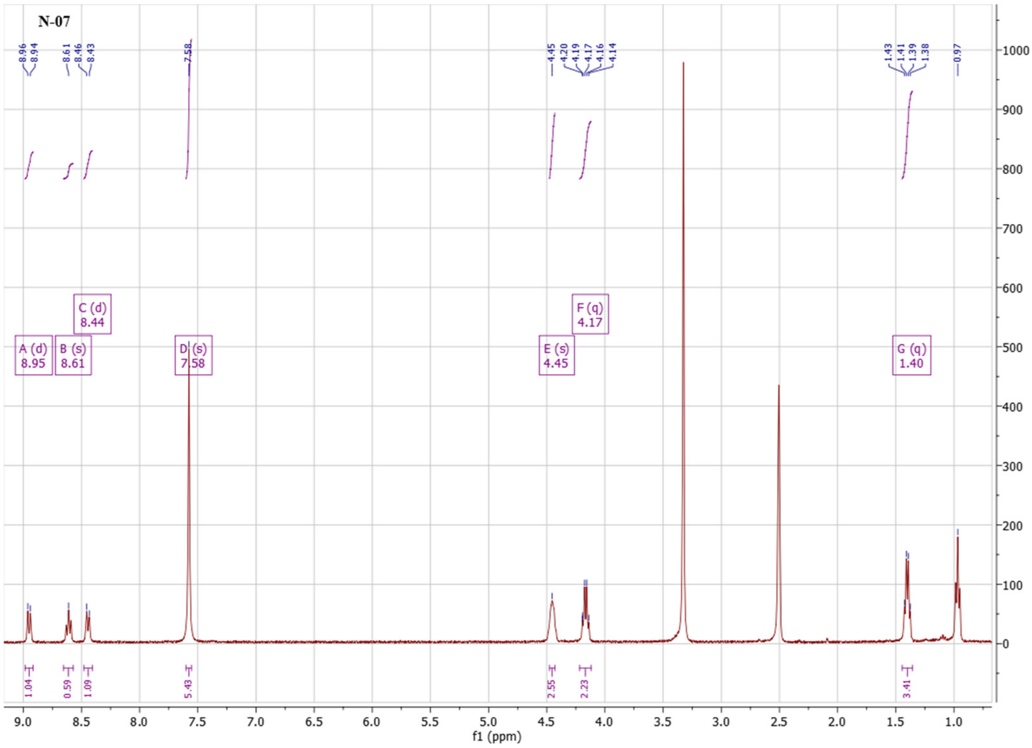
N-07
A (d)
8.95
B (s)
8.61
C (d)
8.44
D (s)
7.58
E (s)
4.45
F (q)
4.17
G (q)
1.40
f1 (ppm)

N-09

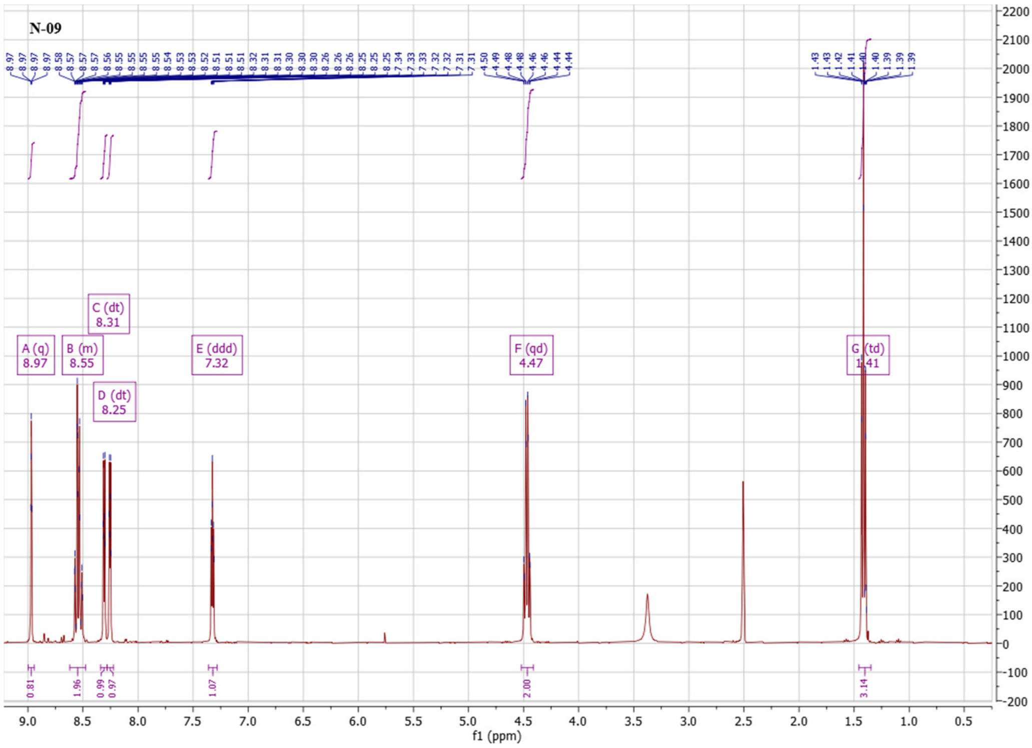
N-09
A (q)
8.97
B (m)
8.55
C (dt)
8.31
D (dt)
8.25
E (ddd)
7.32
F (qd)
4.47
G (td)
1.41
f1 (ppm)

N-10

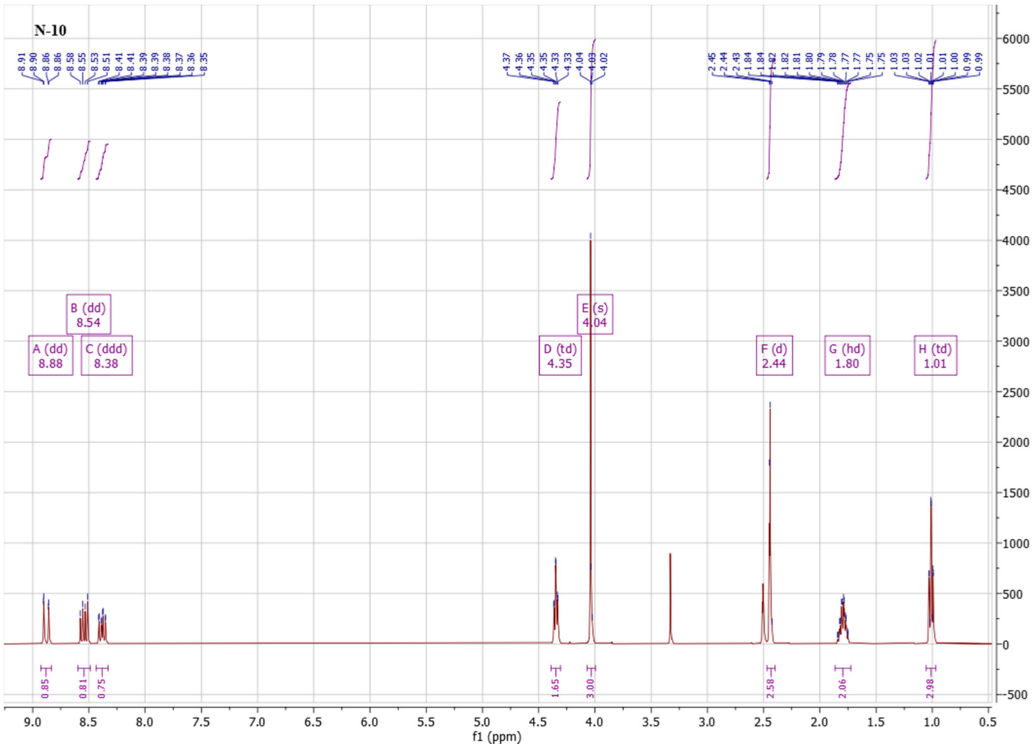
N-10
A (dd)
8.88
B (dd)
8.54
C (ddd)
8.38
D (td)
4.35
E (s)
4.04
F (d)
2.44
G (hd)
1.80
H (td)
1.01
f1 (ppm)

N-11

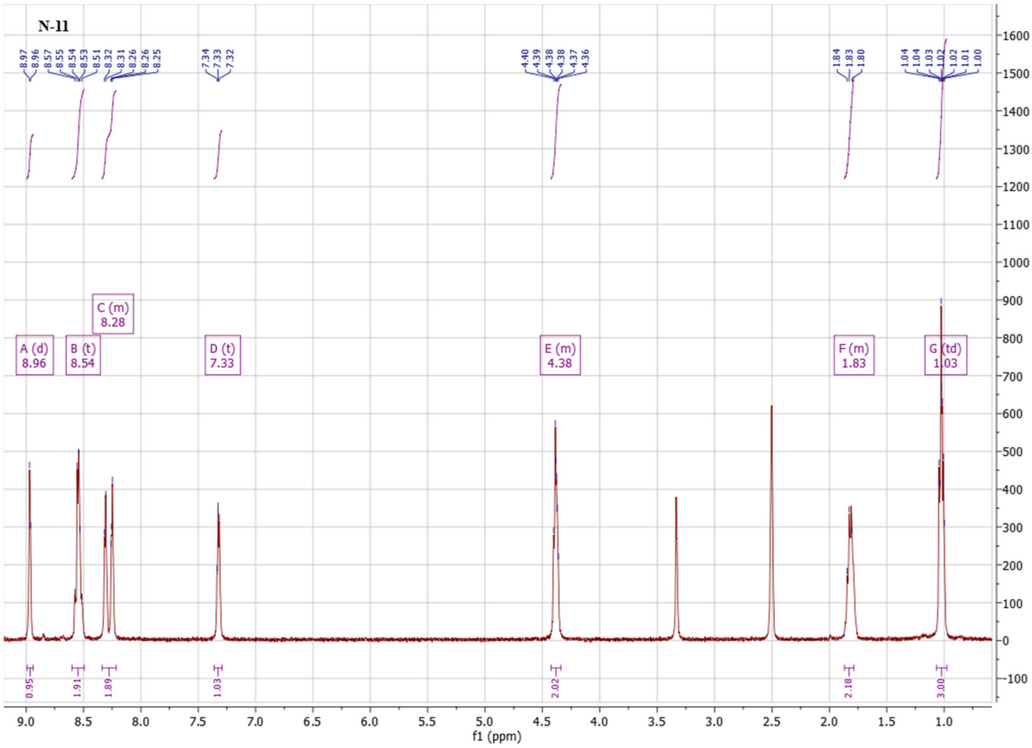
N-11
A (d)
8.96
B (t)
8.54
C (m)
8.28
D (t)
7.33
E (m)
4.38
F (m)
1.83
G (td)
1.03
f1 (ppm)

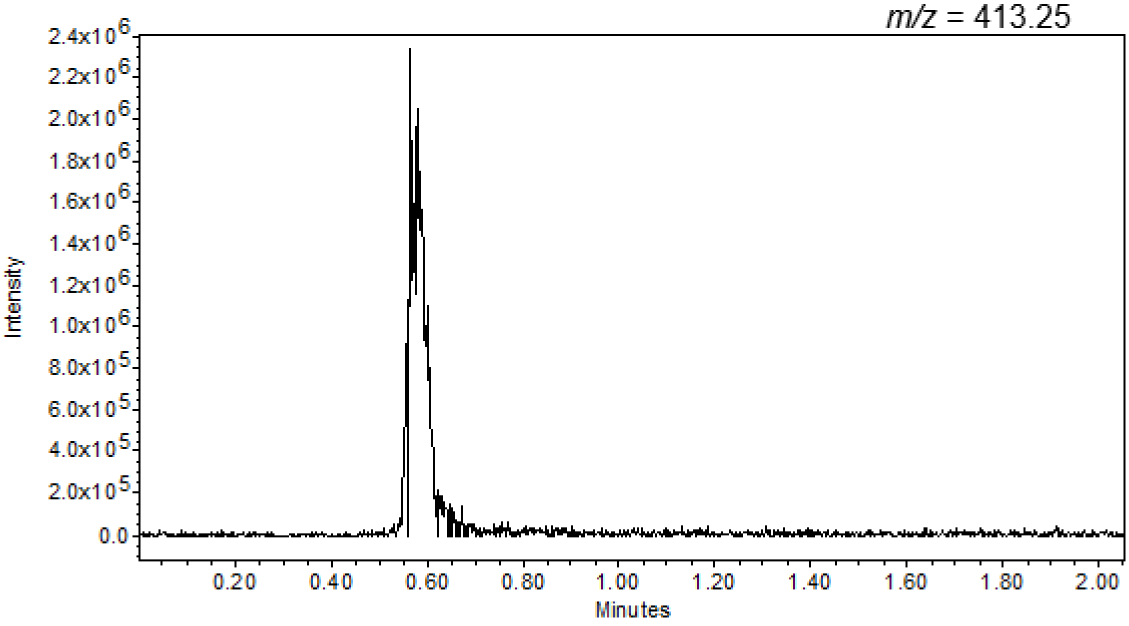

m/z = 413.25
Intensity
Minutes

N-12

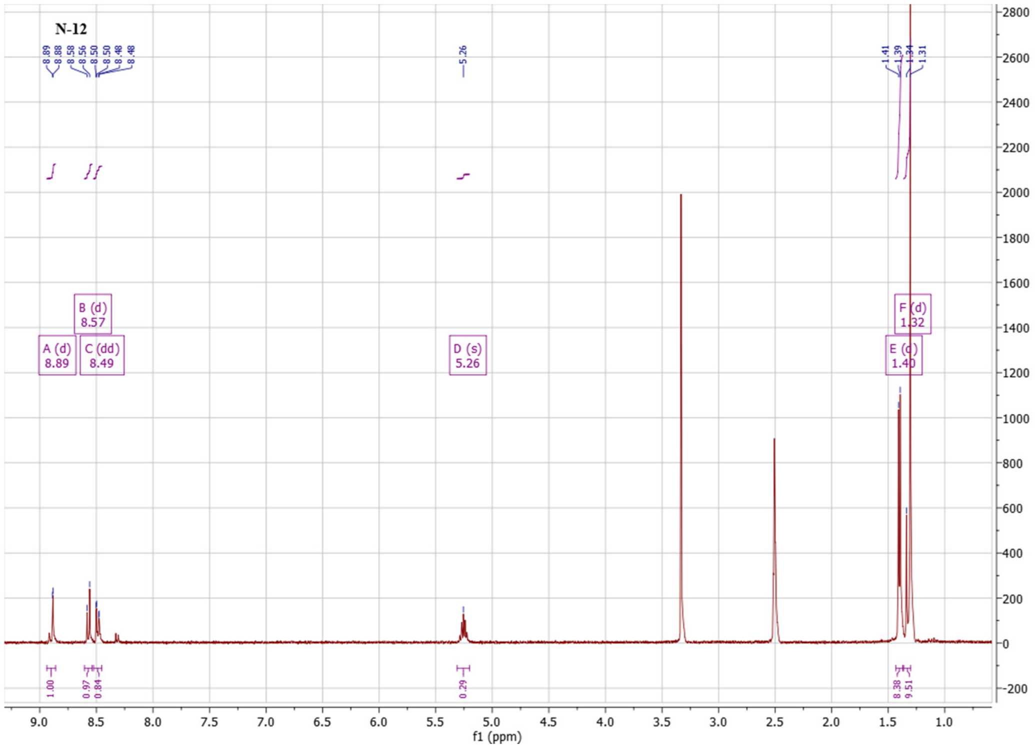

N-12
A (d)
8.89
B (d)
8.57
C (dd)
8.49
D (s)
5.26
E (d)
1.40
F (d)
1.32
f1 (ppm)

N-13

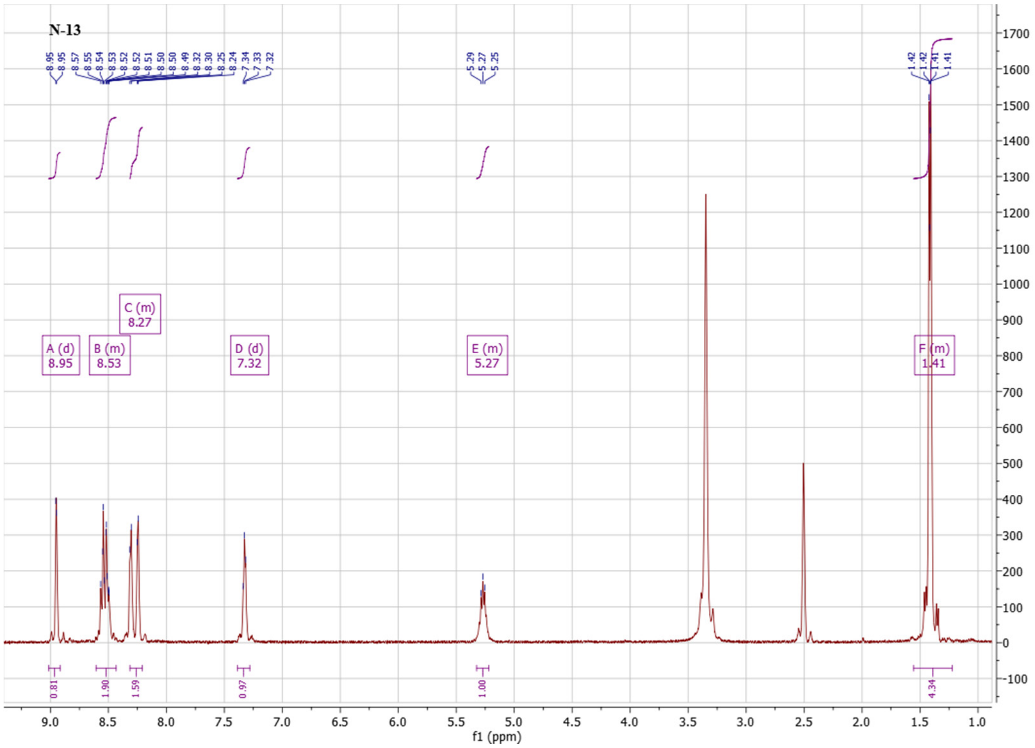
N-13
A (d)
8.95
B (m)
8.53
C (m)
8.27
D (d)
7.32
E (m)
5.27
F (m)
1.41
0.81
1.90
1.59
0.97
1.00
4.34
f1 (ppm)

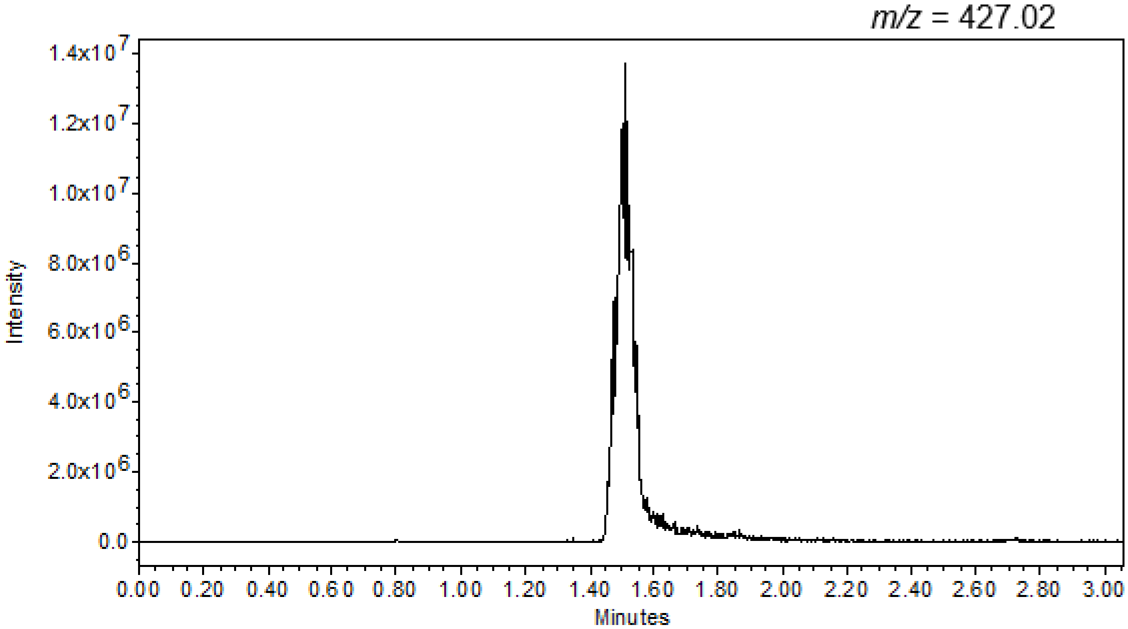
m/z = 427.02
Intensity
Minutes

Comparative chart for the antimycobacterial and antinocardial activities of quinoxaline-1,4-di-*N*-oxide derivatives

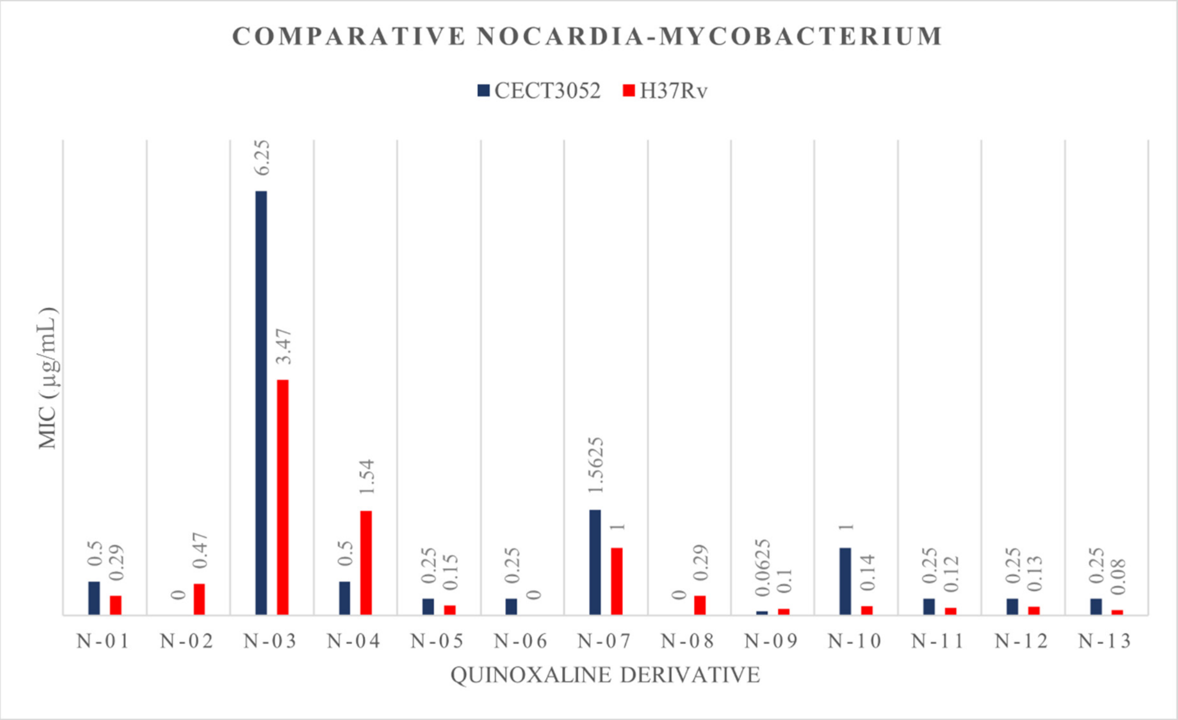

Supplementary Chart 1. Comparison of the newly reported antinocardial activity and previously reported antimycobacterial activity of quinoxaline-1,4-di-*N*-oxide derivatives. *Nocardia brasilensis,* reference strain CECT3052 (blue), and *Mycobacterium tuberculosis* reference stain H37Rv (red), zero values represent not determined MIC values.
